# Anterior Maxillary Ameloblastic Fibroma in a Pediatric Patient: A Case Report

**DOI:** 10.7759/cureus.82918

**Published:** 2025-04-24

**Authors:** Selma Daoudi, Elmehdi Hariri, Hind Ramdi

**Affiliations:** 1 Pediatric Dentistry, Mohammed V University in Rabat, Rabat, MAR; 2 Dentistry, Mohammed V University in Rabat, Rabat, MAR

**Keywords:** ameloblastic fibroma, anterior maxillary, incidental finding, oral tumor, pediatric patient

## Abstract

Ameloblastic fibroma (AF) is a rare benign mixed odontogenic tumor, more frequently observed in children and adolescents. It typically develops in the posterior region of the mandible. Radiographically, it appears as a well-defined radiolucent lesion, requiring histological confirmation to differentiate it from other odontogenic tumors.

We report the case of an eight-year-old male patient in good general health who presented following an anterior trauma. The extraoral examination was normal, while the intraoral examination revealed the avulsion of tooth 11, a complicated enamel-dentin fracture of tooth 21, and a firm palatal swelling. Radiographic evaluation showed a unilocular radiolucent lesion containing radiopaque structures, initially suggesting a diagnosis of compound odontoma. Complete enucleation of the lesion revealed calcified components and soft tissue. Histopathological analysis confirmed the diagnosis of AF.

A conservative enucleation was performed due to the well-defined margins of the lesion, along with apexification of the adjacent permanent tooth 21. A six-month postoperative follow-up showed satisfactory bone healing with no signs of recurrence. Given that this fibroma presents a potential for recurrence and malignant transformation, rigorous long-term follow-up is essential. This case highlights the importance of thorough clinical, radiographic, and histological evaluation of jaw lesions in pediatric patients, as well as the necessity of long-term follow-up to ensure early detection of any complications.

## Introduction

According to the World Health Organization (2022), benign odontogenic tumors are classified into three major categories based on their histogenetic origin: epithelial, mesenchymal, and mixed. A notable update in this classification is the inclusion of adenoid ameloblastoma as a new entity.

Ameloblastic fibroma (AF) is a rare benign mixed odontogenic tumor characterized by both epithelial and mesenchymal components, and is grouped with lesions such as odontoma, primordial odontogenic tumor, and dentinogenic ghost cell tumor [1]. It represents only 1.5% to 4.5% of all odontogenic tumors, predominantly affecting children and adolescents, with 75% of cases diagnosed before the age of 20. It has a male-to-female ratio of 1.4:1 [2,3].

The posterior region of the mandible is the most frequently affected site. Clinically, these tumors are often asymptomatic and may be discovered incidentally during routine dental radiographs. However, some patients may present with painless swelling of the jaw. Radiographically, they appear as well-defined radiolucent lesions, either unilocular or multilocular, and are often surrounded by sclerotic margins [4]. The unilocular variants present as asymptomatic cases, whereas multilocular variants are more likely to cause noticeable jaw enlargement or swelling [5]. Given their imaging characteristics, several other jaw lesions must be considered in the differential diagnosis, including ameloblastoma, dentigerous cyst, odontogenic keratocyst, central giant cell granuloma, and histiocytosis. In rare instances where cellular atypia or abnormal mitoses are observed, the possibility of malignant transformation into ameloblastic fibrosarcoma should be explored.

Histologically, AF is composed of a primitive ectomesenchymal tissue resembling dental papilla, interspersed with strands and islands of odontogenic epithelium. The absence of dental hard tissue is a defining feature that differentiates it from other mixed odontogenic lesions. The treatment of choice is conservative surgical removal, often combined with extraction of the associated teeth, followed by long-term clinical and radiographic follow-up to monitor for recurrence or rare malignant transformation [4-6].

## Case presentation

An eight-year-old male patient, with no significant medical history, presented to the Pedodontics Department at the Rabat Dental Consultation and Treatment Center with acute pain localized to tooth 21. His history revealed a traumatic injury three months earlier, resulting in the avulsion of the right maxillary central incisor (tooth 11) and a complicated enamel-dentin fracture of the left maxillary central incisor (tooth 21). Neither the avulsed tooth nor the fractured fragment had been recovered.

**Figure 1:**
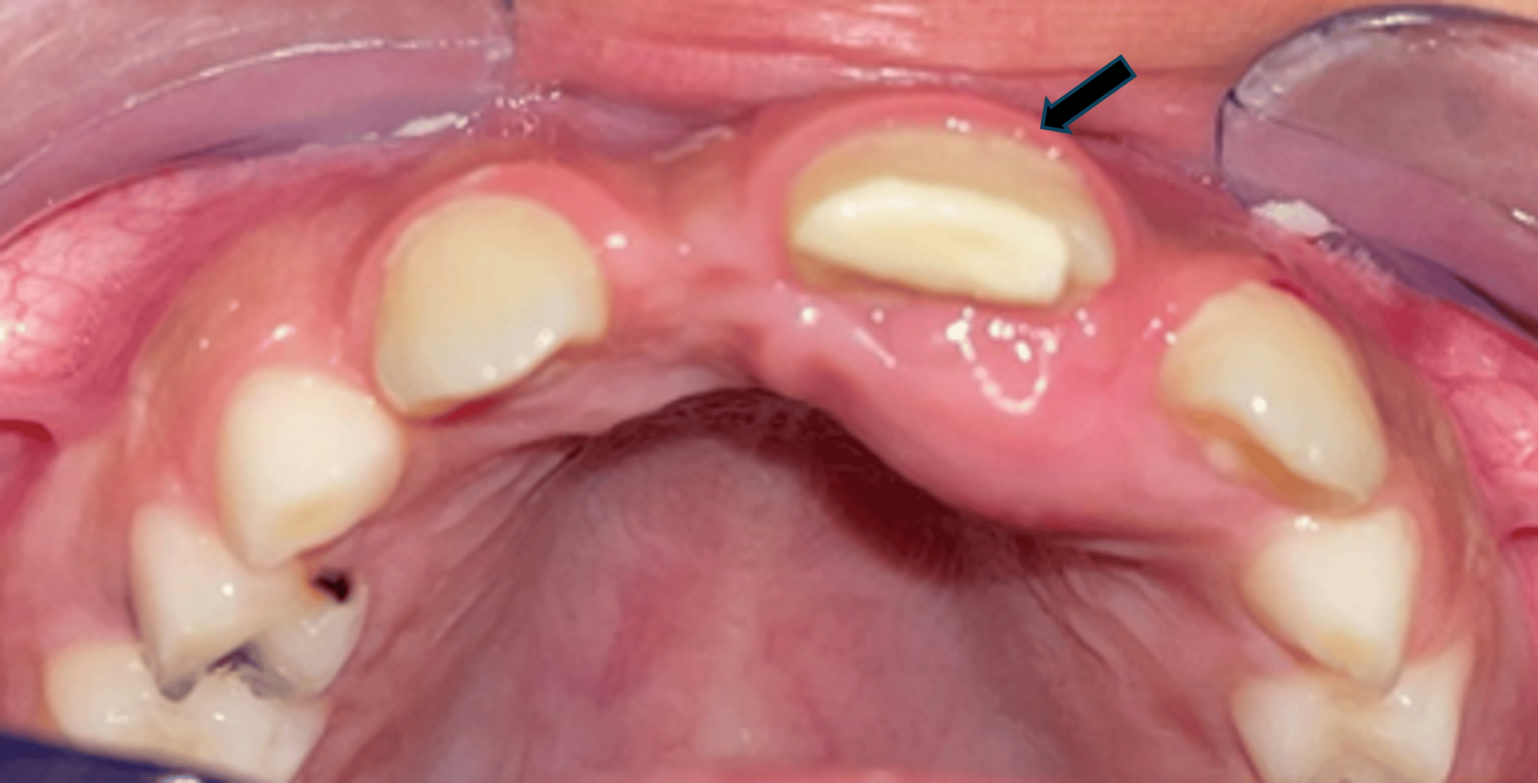
Preoperative clinical image showing the absence of tooth 11 and mesial tilting of tooth 21 (arrow); a palatal swelling is visible between teeth 21 and 22, covered by normal-appearing mucosa.

Extraoral examination revealed no facial asymmetry, cervical lymphadenopathy, or other abnormalities. Intraorally, tooth 11 was missing, and tooth 21 exhibited mesial inclination. A firm, painful swelling was noted on the palatal aspect between teeth 21 and 22, covered by clinically normal palatal mucosa (Figure 1).

According to the patient’s mother, the swelling had been present since the exfoliation of the primary teeth and had gradually increased in size. Cold and percussion testing on tooth 21 elicited hypersensitive responses, indicating pulpal involvement.

**Figure 2:**
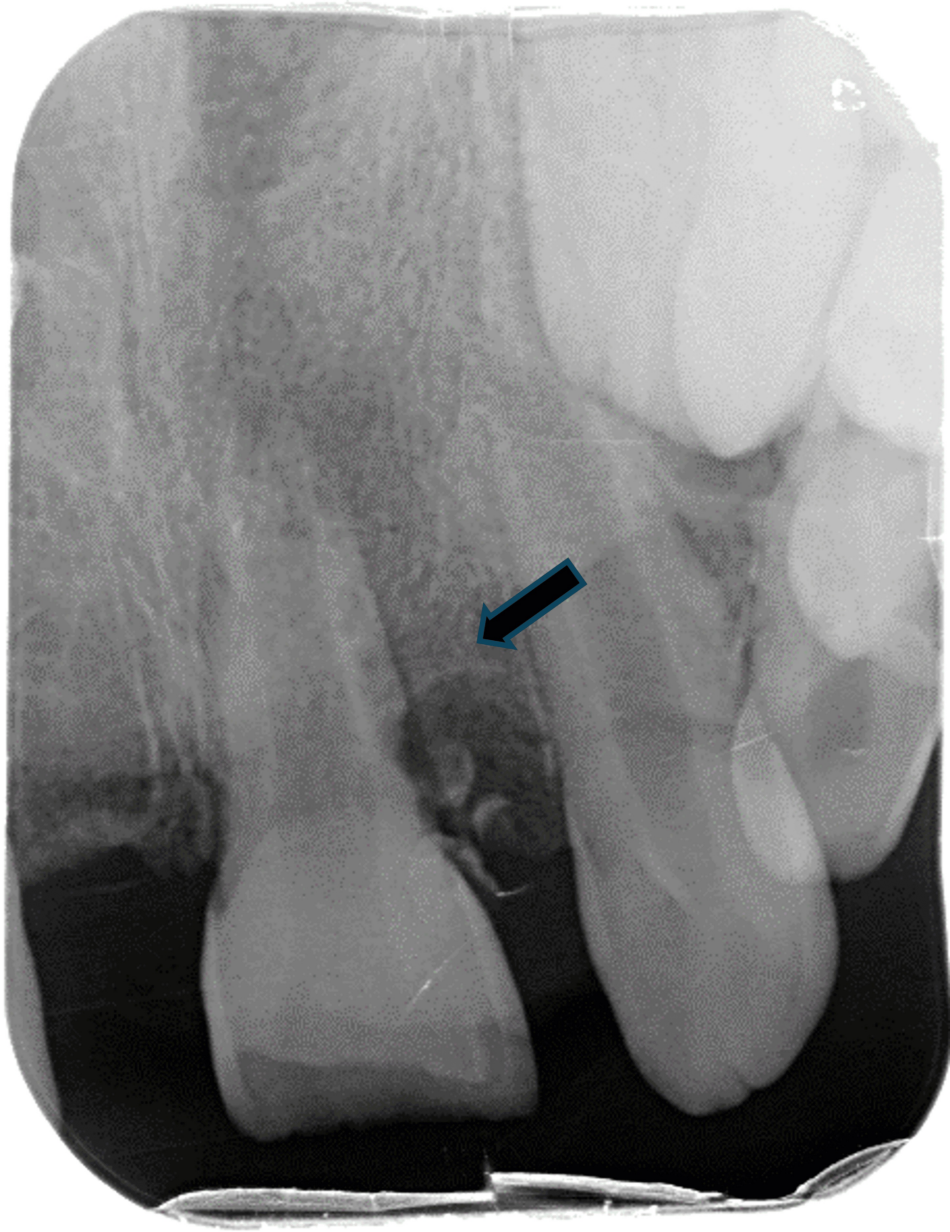
Well-defined unilocular radiolucent lesion (arrow) in the cervical third region between teeth 21 and 22, with two radiopaque elements within it

Radiographic evaluation demonstrated a well-defined unilocular radiolucent lesion in the anterior maxilla, localized in the cervical third between teeth 21 and 22. Within the lesion, two radiopaque structures were visible (Figure 2).

Based on the clinical and radiographic findings, a provisional diagnosis of compound odontoma was made. However, due to its radiographic appearance, the differential diagnosis included other entities such as AF, odontogenic keratocyst, ameloblastoma, and central giant cell granuloma, ameloblastic fibrosarcoma was also considered due to the potential for malignant transformation, especially in the presence of mitotic figures or cellular atypia.

**Figure 3:**
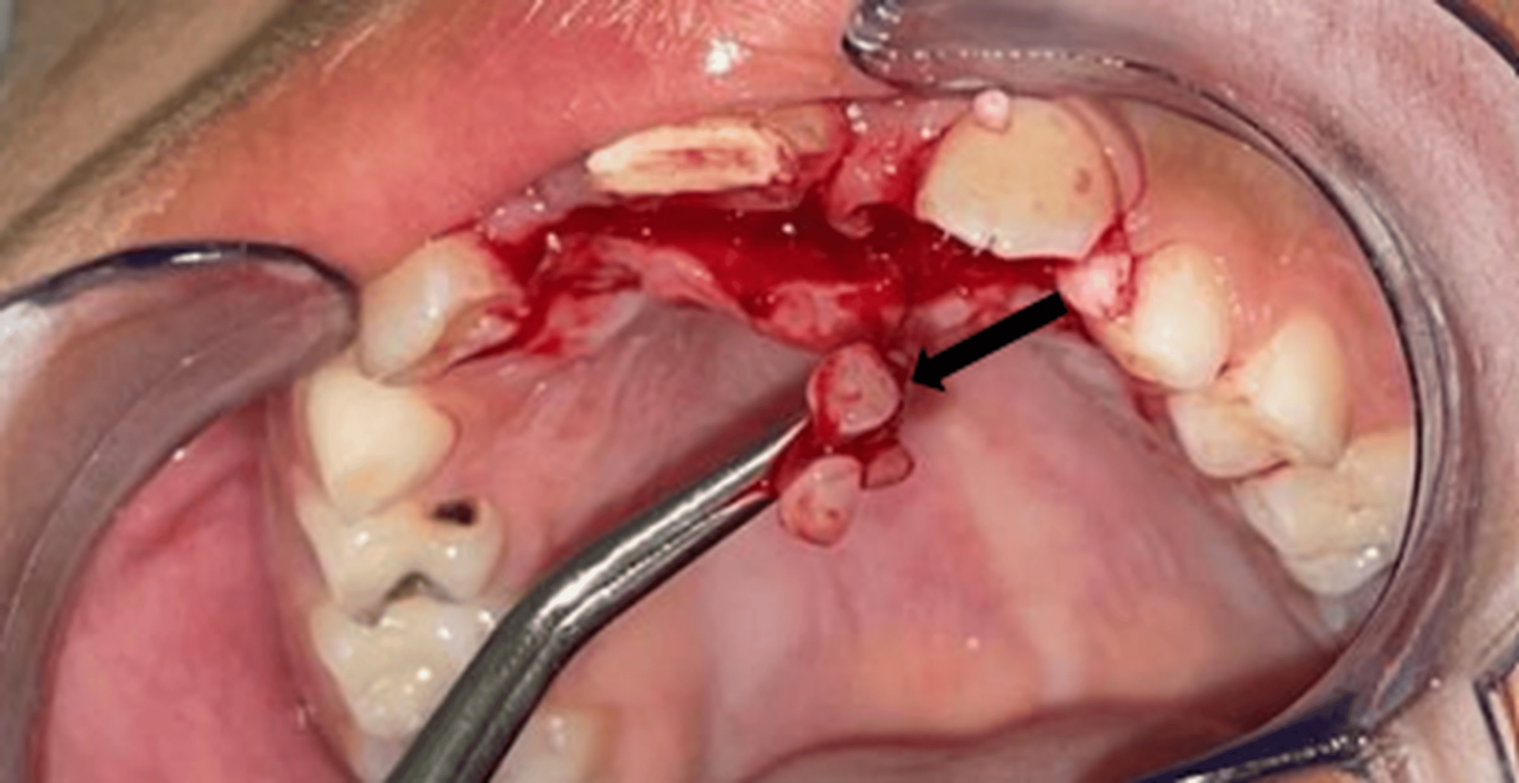
Intraoperative view showing calcified structures resembling small tooth-like formations (arrow), extracted from the lesion

**Figure 4:**
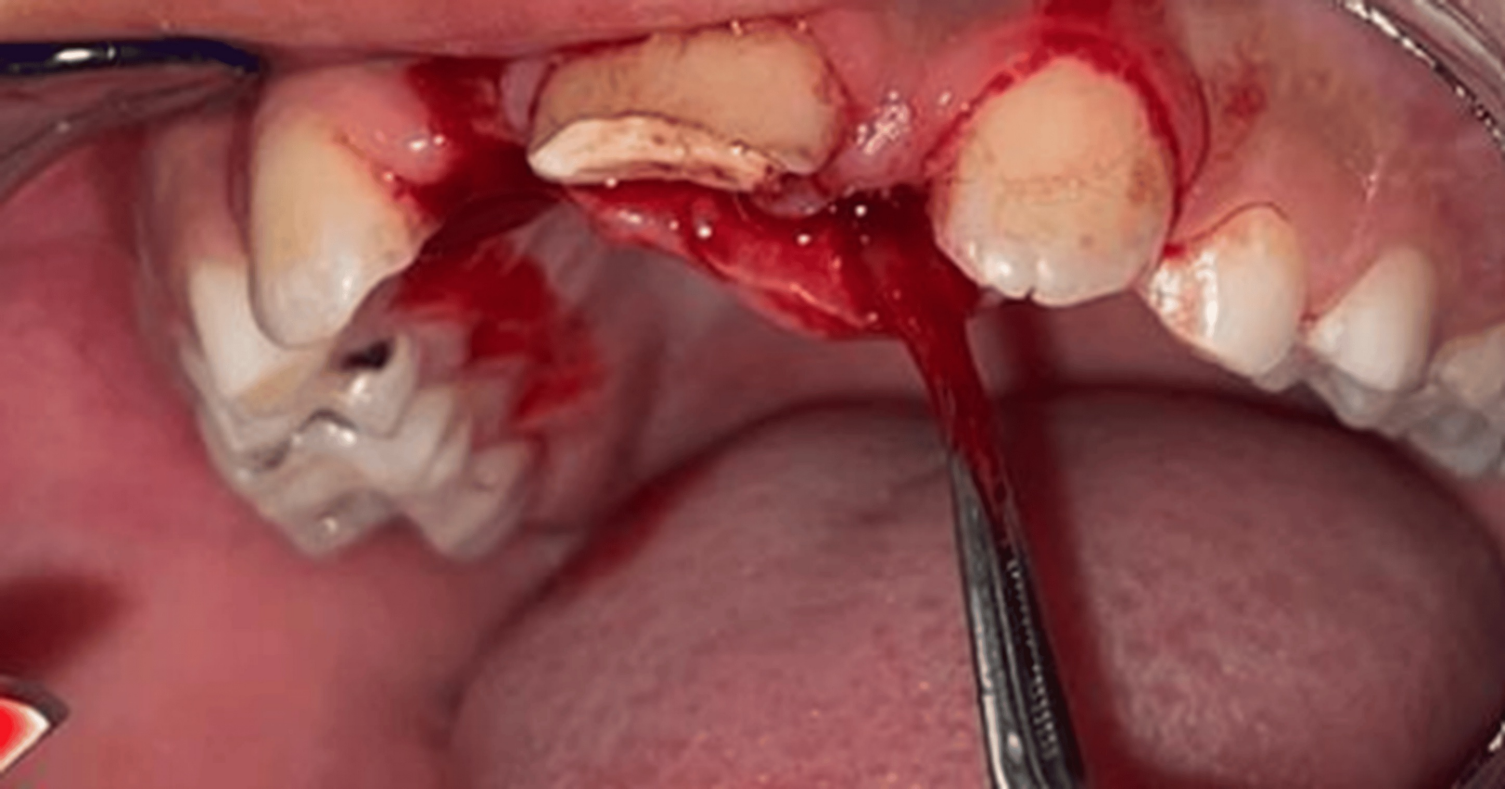
Soft tissue fragment with a follicular-like appearance, suggestive of an odontogenic origin

The treatment plan involved complete enucleation of the lesion through a palatal flap approach, performed as an excisional biopsy. The procedure revealed both tooth-like calcified material and soft tissue resembling a follicular sac (Figures 3, 4).

**Figure 5:**
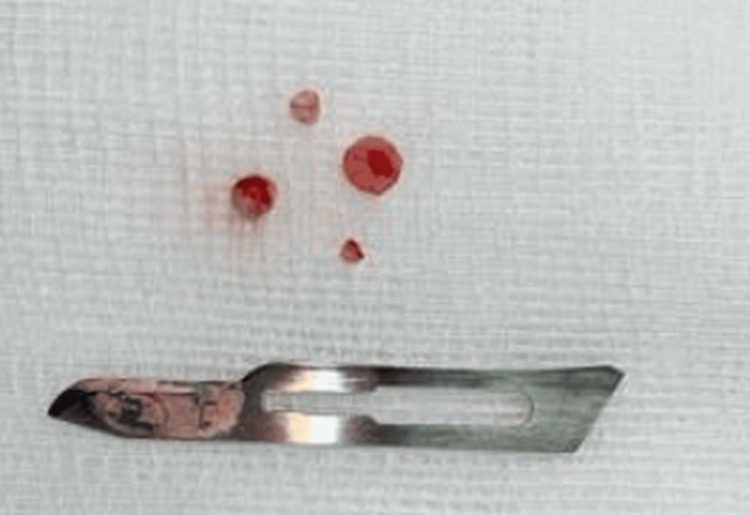
Extracted calcified tissues from the lesion

The calcified material was photographed and shown in Figure 5.

**Figure 6:**
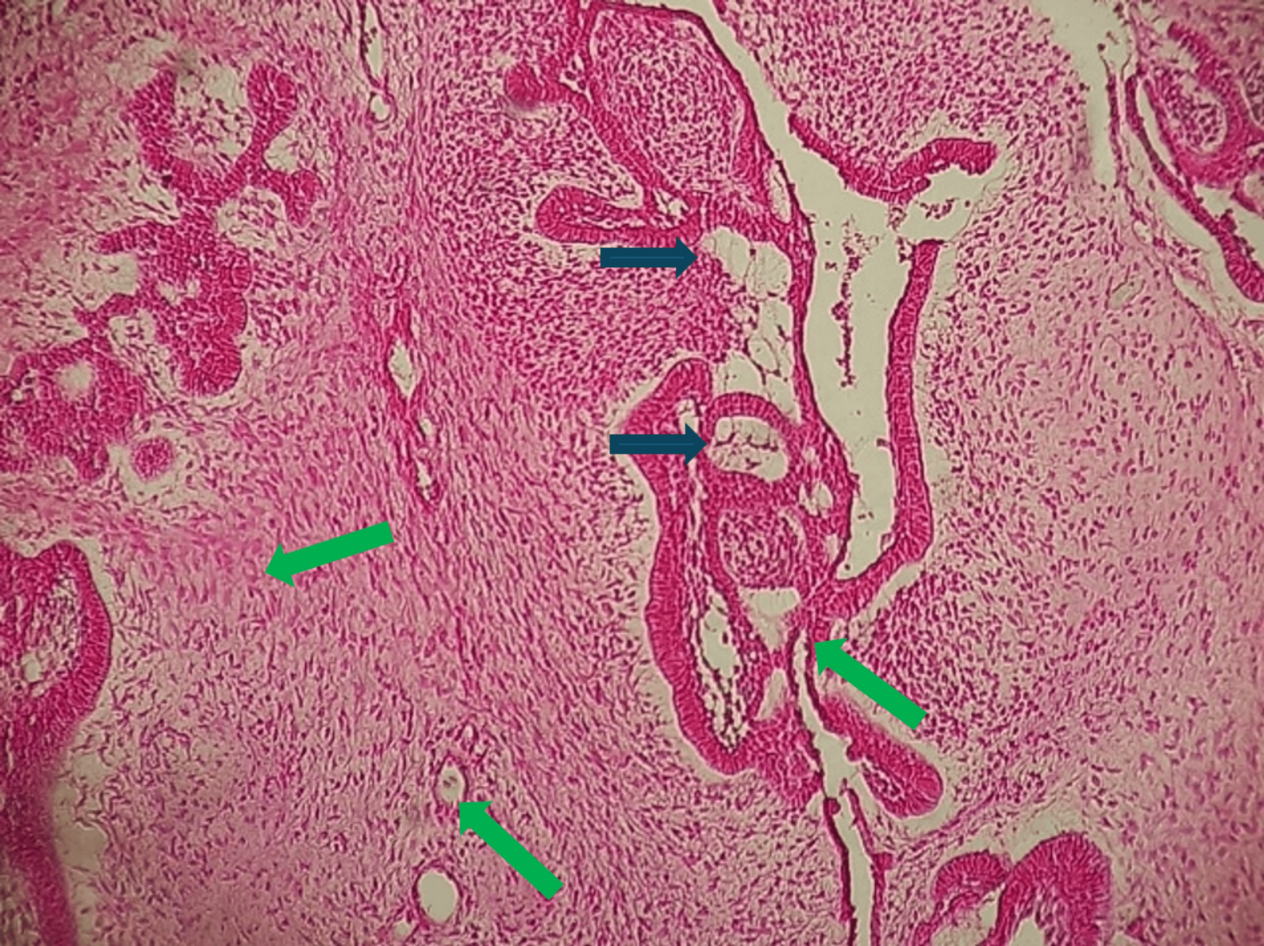
Histological section of ameloblastic fibroma (HEX10) The epithelial component is represented by follicles of odontogenic epithelium (black arrows), while the mesenchymal stroma resembles dental papilla tissue (green arrows). HEX10: at 10X magnification

**Figure 7:**
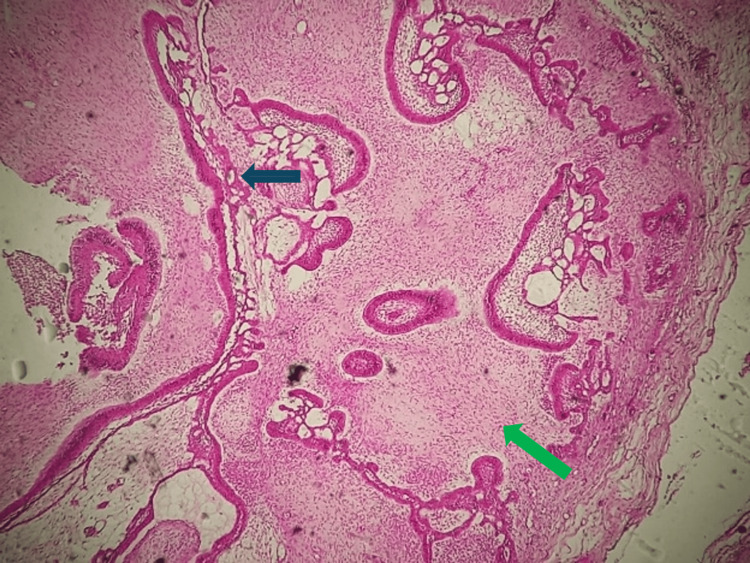
Histological section of ameloblastic fibroma (HEX20) The odontogenic epithelial component is organized into follicular islands (black arrow), while the surrounding mesenchymal tissue resembles dental papilla (green arrow). No cytonuclear atypia is observed. HEX20: at 20X magnification

Histopathological examination revealed a neoplasm composed of proliferating odontogenic epithelium embedded in a cellular mesenchymal tissue resembling dental papilla, consistent with AF (Figures 6, 7).

The epithelial component exhibited strands and islands of columnar or cuboidal cells, without atypia or mitotic activity. The mesenchymal component was composed of a loose connective tissue stroma with stellate and spindle-shaped cells. No calcified dental tissue or malignant features were noted. Although routine hematoxylin and eosin (H&E) staining was sufficient for diagnosis in this case, immunohistochemical staining may assist in difficult cases. Immunomarkers such as cytokeratin for epithelial cells, vimentin for mesenchymal components, and Ki-67 for proliferative activity could help differentiate benign lesions from malignant ones such as ameloblastic fibrosarcoma. However, given the typical histologic features and absence of atypia, further staining was not deemed necessary.

**Figure 8:**
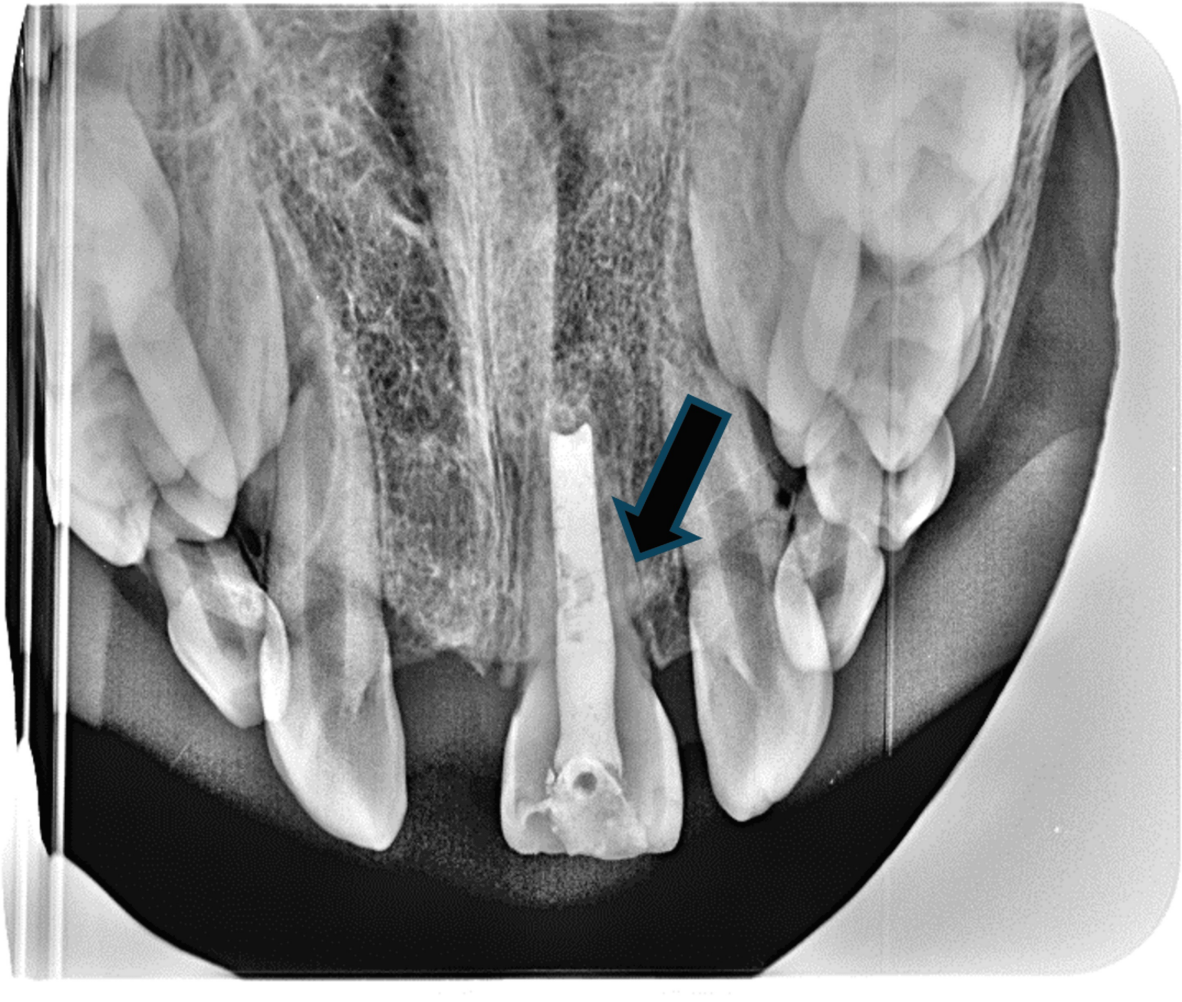
Occlusal radiograph after six months showing complete healing with tooth 21 treated by apexification (arrow)

**Figure 9:**
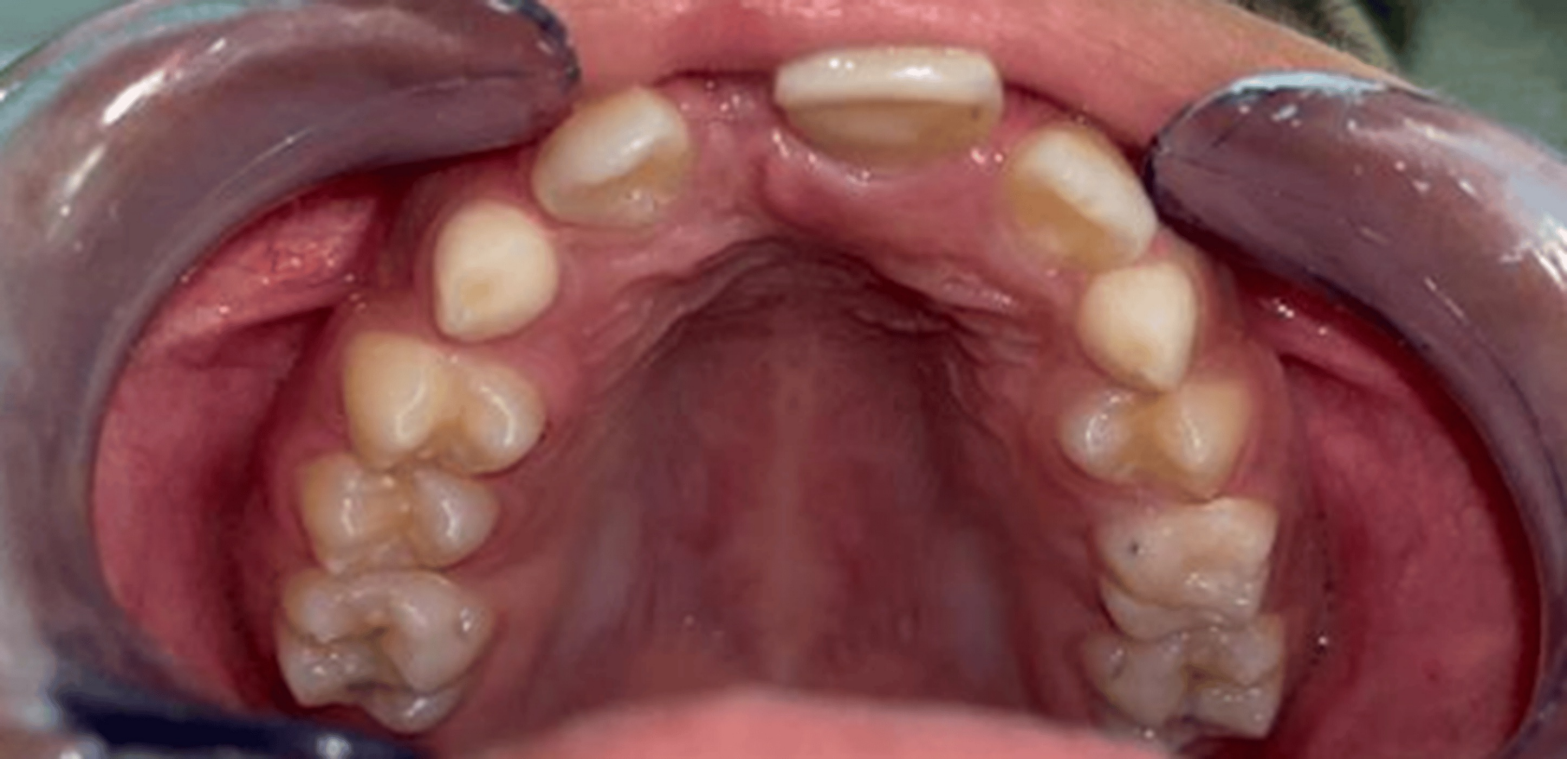
Endobuccal image after six months of healing

Endodontic management of tooth 21 was initiated with apexification using Biodentine (Septodont, Saint-Maur-des-Fossés, France), followed by root canal obturation with warm gutta-percha. Radiographic follow-up showed satisfactory bone healing, and clinical follow-up after six months demonstrated healthy gingival healing (Figures 8, 9).

Continued postoperative monitoring was advised to detect any potential recurrence.

## Discussion

AF is a rare odontogenic tumor, often associated with impacted teeth (75% of cases) and sometimes discovered incidentally on radiographs (20% of cases). It primarily affects the posterior mandible (85-90%), with maxillary occurrences being rare. The tumor size ranges from 1 to 10 cm, with over half exceeding 4 cm. It predominantly arises in children and adolescents, mostly in the first two decades of life, with a slight male predominance [7-9].

The pathogenesis of AF remains controversial, as it resembles early tooth development before hard tissue formation. Some theories suggest that AF follows a developmental pattern like the tooth germ, but its exact mechanisms remain unclear. The epithelial cells in AF appear too primitive to induce odontoblastic differentiation, and the reason for this remains unexplained. Cahn and Blum’s maturation theory proposes that AF differentiates progressively into ameloblastic fibro-odontoma (AFO) and eventually into odontoma. However, this theory is not widely accepted, as recurrent AF does not typically develop into hard tissue tumors, and it often appears after odontogenesis is complete [5,10].

AF is a painless, slow-growing, and expansive tumor, as demonstrated by the case reported in this article. Unlike simple ameloblastoma, AF exhibits slower clinical growth and does not infiltrate between the bone trabeculae. Instead, it enlarges gradually while typically maintaining a smooth periphery. In most cases, it remains asymptomatic, with swelling being the most common clinical sign. However, in our case, the diagnosis was made incidentally after the patient sought consultation due to the trauma rather than a noticeable swelling. A particularly noteworthy aspect of our case is the unusual location of the AF, in the anterior maxilla. While this localization is extremely rare, it has been previously reported by McGuinness et al. [11]. An AF is most commonly found in the posterior mandible, and its occurrence in the anterior maxilla remains exceptional, further expanding the known spectrum of this rare entity. Another striking feature of our case is the presence of calcified tissue, a characteristic rarely described in AF. The literature indicates that calcifications are more commonly associated with calcifying odontogenic cysts (COC) rather than AF. In our case, areas of calcified material were observed within the lesion, an uncommon finding that further distinguishes this case. This atypical presence of calcification highlights the histopathological variability of the tumor and suggests potential overlapping characteristics with other odontogenic lesions [7,12].

Radiographic examination of the tumor may reveal a unilocular or multilocular lesion that expands the cortical plates of the mandible, and it is usually characterized by well-circumscribed borders. In rare cases, the borders may be scalloped, and root resorption may be present [13,14].

Histological variants of AF documented in the literature include granular cell AF, characterized by granular cells in the ectomesenchyme, papilliferous AF, which shows significant epithelial proliferation with a plexiform arrangement, and cystic AF. Some cases of ameloblastoma associated with AF have also been reported [15]. Histopathologically, our case presented the classic features of AF, with strands, cords, and islands of odontogenic epithelium embedded in richly cellular ectomesenchyme. The epithelial component consisted of double-layered strands of cuboidal cells and islets bordered by tall cylindrical cells with polarized nuclei, surrounding stellate cells reminiscent of the star reticulum. Juxta-epithelial hyalinization was present around some epithelial islets, with perifollicular areas devoid of clear cells. Microphotographs showed dual epithelial and stromal proliferation (HEX10). The epithelial component consisted of ameloblastic cells organized in a follicular structure, while the moderately cellular stromal component showed no cytonuclear atypia (HEX20).

Distinguishing AF from other odontogenic lesions can be difficult, as its soft tissue component closely resembles the developing dental papilla, with the presence of epithelial strands and little or no induction of dental hard tissue. However, in our case, we observed the presence of calcified hard tissue, an unusual feature in AF, which may complicate differential diagnosis with other odontogenic tumors such as ameloblastic fibrodentinoma (AFD), ameloblastic fibro-odontoma (AFO), and even odontomas in their early stages. Additionally, central odontogenic fibroma and odontogenic myxoma should be considered when dealing with radiolucent lesions of similar appearance. Histological examination remains essential to distinguish between these entities [3,13,14].

Treatment of AF consists mainly of complete enucleation of the tumour, often accompanied by removal of the impacted teeth and curettage of the peripheral bone. This approach is favored because of the benign nature of the lesion and its well-circumscribed growth, unlike ameloblastoma, which infiltrates bone trabeculae. In our case, given the absence of cortical perforation, the well-defined limits of the lesion, and its non-invasive nature, we opted for a conservative approach with complete enucleation. However, as maxillary involvement has been associated with a higher risk of recurrence, long-term monitoring is necessary. Reported recurrence rates range from 18% to 43.5% [16], with some cases progressing to ameloblastic fibrosarcoma, underlining the need for prolonged postoperative surveillance.

In this case, clinical and radiographic assessments six months after surgery showed satisfactory healing with no signs of recurrence. Nevertheless, given the risk of late relapse or malignant transformation, ongoing follow-up remains crucial to ensure early detection of any complications [16,17].

In summary, this case represents a rare occurrence of AF in the anterior maxilla, an exceptional localization given its high predilection in the posterior mandible. This case is further distinguished by the presence of calcified tissue, an uncommon finding in AF, which complicates the differential diagnosis with other odontogenic tumors such as ameloblastic fibrodentinoma and ameloblastic fibro-odontoma. The atypical localization and histopathological features observed in our case broaden the known spectrum of FA and underline the importance of considering these rare presentations in clinical practice.

## Conclusions

AF is a rare neoplasm that should be considered in the differential diagnosis of extensive jaw lesions, particularly in younger individuals. In our case, the tumor was located in the anterior maxilla and was not associated with an impacted tooth, emphasizing the need for thorough clinical and radiographic evaluation regardless of tooth involvement. Given the patient's age and the tumor's occurrence during the period of odontogenesis, some might consider AF as a non-neoplastic hamartomatous lesion. However, based on current evidence and the clinical presentation of this case, we regard it as a true neoplasm rather than a hamartoma. Careful treatment planning is essential, considering the risk of recurrence and the potential for malignant transformation, underscoring the importance of long-term follow-up.
